# A timed Phalen’s test predicts abnormal electrophysiology in carpal tunnel syndrome

**DOI:** 10.1002/brb3.2056

**Published:** 2021-01-29

**Authors:** Mohammed H. Alanazy, Hana Albulaihe, Ziad Alhumayyd, Anas M. Albarrak, Ahmad R. Abuzinadah

**Affiliations:** ^1^ Department of Internal Medicine College of Medicine King Saud University Riyadh Saudi Arabia; ^2^ Department of Internal Medicine College of Medicine Prince Sattam Bin Abdulaziz University Alkharj Saudi Arabia; ^3^ Department of Internal Medicine King Abdulaziz University Hospital and College of Medicine, King Abdulaziz University Jeddah Saudi Arabia

**Keywords:** carpal tunnel syndrome, CTS, nerve conduction study, Phalen test, positive predictive value, sensitivity, specificity

## Abstract

**Objective:**

Previous studies reported variable sensitivity and specificity of the Phalen test. We investigated whether a timed Phalen’s test (TPT) could predict abnormal nerve conduction studies (NCS) results in carpal tunnel syndrome (CTS).

**Methods:**

Patients with CTS were consecutively recruited. A neurologist confirmed the clinical diagnosis of CTS and recorded the TPT before NCS were performed. Another neurologist, blinded to the TPT, graded the severity of NCS. The TPT sensitivity, specificity, positive predictive value (PPV), and negative predictive value (NPV) were calculated.

**Results:**

In total, 403 patients with 706 hands were recruited and diagnosed with CTS; 465 hands had positive TPT, and 611 hands showed abnormal NCS results. A positive TPT at ≤ 10 s had a specificity of 96.8% and a PPV of 96.6% in predicting abnormal NCS. The sensitivity and NPV of TPT were insignificant.

**Discussion:**

A positive TPT at ≤ 10 s can be useful in predicting NCS abnormalities in patients with CTS.

## Graphical abstract

The timed Phalen’s test can be useful in predicting abnormal NCS that confirms median neuropathy at the wrist in a subgroup of CTS patients.

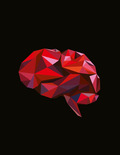

**Abbreviations**
CTS: carpal tunnel syndrome
FPR: false‐positive rate
NCS: nerve conduction studies
NPV: negative predictive value
PPV: positive predictive value
TPT: timed Phalen’s test

## 1 INTRODUCTION

The diagnosis of carpal tunnel syndrome (CTS) is based on clinical features and confirmed by nerve conduction studies (NCS). The sensitivity and specificity of NCS exceeds 85% and 95%, respectively (Jablecki et al., 2002). It has been suggested that physicians incorporate NCS along with clinical measures when assessing patients with CTS (Schrijver et al., 2005). One of these clinical tools is the wrist‐flexion test described by Phalen in 1951 (Phalen, 1951, 1970; Phalen & Kendrick, 1957). A false‐positive Phalen test has been reported in 20% of normal subjects as well as in other conditions including cervical radiculopathy, tendonitis, and ulnar neuropathy (Jablecki et al., 2002). Subsequent studies revealed a wide range of sensitivity, ranging from 42% to 85%, and specificity, ranging from 54% to 98% (Brüske et al., 2002; D'Arcy & McGee, 2000). This variability limits the diagnostic utility of the Phalen test (D'Arcy & McGee, 2000). However, previous studies have not thoroughly explored the rapidity of symptoms provocation by the Phalen test in relation to NCS findings.

This study sought to assess whether a timed version of the Phalen test (TPT) can predict abnormal NCS results in patients diagnosed with clinical CTS. If successful, this simple clinical tool could be used to predict NCS abnormalities, possibly decrease the burdens of long waiting lists and high costs, and mitigate the unavailability of NCS machines and expertise in some areas of the world.

## 2 MATERIALS AND METHODS

### 2.1 Patient inclusion

We recruited adult patients clinically diagnosed with CTS consecutively from the neurophysiology units at King Saud University Medical City and King Abdulaziz University Hospital, located respectively in Riyadh and Jeddah, Saudi Arabia. The study was approved by the institutional review board at both centers. The study commenced in January 2016 and ended in April 2020. Referrals were received from both primary care and specialized clinics. CTS was defined by meeting all the following criteria: (a) numbness, paresthesia, and/or pain in at least two median‐innervated digits for at least 30 days; (b) the symptoms must have been triggered by manual activities, awakened patients from sleep or presented upon awakening at least once in the previous 2 weeks, and (c) the symptoms must have been relieved by shaking of the hands, changing their position, or using a wrist splint. We included patients who met these criteria but could not, by medical history, assure paresthesia/numbness sparing of the ulnar‐innervated digits. We excluded patients who presented with continuous hand numbness/paresthesia, clinical features of severe polyneuropathy, cervical radiculopathy, or had undergone carpal tunnel release surgery. We also excluded patients in whom neurological signs extended beyond the distribution of the median nerve, such as sensory loss in the thenar eminence, little finger, proximal to the wrist, or weakness in ulnar‐, radial‐, or forearm median‐innervated muscles. All patients signed informed consent.

### 2.2 Clinical assessment

A neurologist assessed patients for inclusion and exclusion criteria, recorded the TPT, and completed a data collection form before NCS were performed. Patients were asked to flex both wrists by placing the dorsal surface of the hands against each other while the elbows were flexed and arms were abducted. Wrist flexion was maintained at the maximum possible degree up to 90° for 60 seconds (s), or until positive results were reported. A positive TPT was defined as numbness or paresthesia in at least one of the median‐innervated digits. Patients were instructed to immediately inform the examiner once the symptoms began. The examiner recorded the time at which patients reported the onset of symptoms for each hand.

### 2.3 Electrodiagnosis of abnormal nerve conduction

Two trained technicians blinded to TPT results performed NCS following the protocol described previously (Alanazy, 2017). In brief, antidromic sensory NCS were conducted for median digits 2 and 4, and ulnar digits 4 and 5. Both nerves were stimulated 14 cm proximal to the recording electrode. Palmar orthodromic mixed studies were conducted by stimulating median and ulnar nerves in the palm and recording the respective nerve 8 cm proximal to the stimulation site. Motor NCS were conducted by using a belly‐tendon montage and stimulating the respective nerve 7 cm proximal to the recording electrode. Another neurologist blinded to the TPT graded the NCS on a 6‐point scale modified from Padua et al. (Alanazy, 2017; Padua et al., 1997) Grade 0 indicates normal NCS; grade 1 (minimal) indicates abnormalities in at least two comparison studies (Palmdiff ≥ 0.3 ms, ringdiff ≥ 0.5 ms, median digit 2 vs ulnar digit 5 peak latency difference ≥ 0.5 ms); (Werner & Andary, 2011) grade 2 (mild) indicates slowed median sensory conduction velocities; grade 3 (moderate) indicates prolonged distal motor latency > 4.3 ms; grade 4 (severe) indicates decreased median compound muscle action potential amplitudes < 4.5 mV and absent or decreased sensory nerve action potential amplitudes; grade 5 (extreme) indicates the absence of motor and sensory potentials.

### 2.4 Analysis

Spearman correlation coefficients were used to assess the relationship between TPT, NCS severity, age, and diabetes. NCS results were dichotomized into normal (grade 0) and abnormal (grades 1–5). Sensitivity, specificity, positive predictive value (PPV), and negative predictive value (NPV) of a positive TPT at ≤10 , 15, 20, 25, 30, 35, 40, 45, 50, 55, and 60 s in predicting abnormal NCS were calculated. PPV indicates the likelihood that a positive TPT at each cutoff value would predict abnormal NCS. Negative predictive value indicates the likelihood that a negative TPT at each cutoff value would predict a normal NCS. Two‐tailed *p* < .05 were considered significant. Data were analyzed using the software SPSS (version 23, Chicago, IL).

## 3 RESULTS

In total, 403 patients (81.1% women, 18.9% men) with a mean age of 51.6 ± 11.5 years were recruited. Among the participants, 377 (93.5%) were right‐handed, and 130 (32.3%) had diabetes. There were a total of 706 hands with CTS (303 bilateral, 64 right hand only, and 36 left hand only), 611 (86.5%) of which displayed abnormal NCS. The mean TPT was 24.1 ± 13.6 s. The sensitivity, specificity, PPV, and NPV of each TPT cutoff value are shown in Table 1. A positive TPT at ≤10 s had a high specificity (96.8%) and PPV (96.6%). These results were essentially identical when the right‐ and left‐hand data were analyzed separately (Supplementary Material). Weak correlations were observed between age and NCS severity (rho = 0.34, *p* < .001). The correlation coefficient between the TPT and NCS severity was – 0.13 (*p* = .006). No significant correlations were observed between the TPT and age or diabetes (Supplementary Material). The distribution of NCS severity grading across the different TPT cutoff values is shown in Table 2.

**TABLE 1:** Diagnostic accuracy of the timed Phalen’s test in predicting abnormal NCS results in patients diagnosed with CTS (*N* = 706 hands)

Phalen time (s)	Hands, *N* (%)	Sensitivity (%)	Specificity (%)^a^	Positive predictive value (%)	Negative predictive value (%)
≤10	88 (12.5)	13.9	96.8	96.6	14.9
≤15	158 (22.4)	24.2	89.5	93.7	15.5
≤20	224 (31.7)	34.4	85.3	93.8	16.8
≤25	283 (40.1)	43.5	82.1	94.0	18.4
≤30	342 (48.4)	51.4	70.5	91.8	18.4
≤35	365 (51.7)	54.5	66.3	91.2	18.5
≤40	400 (56.7)	59.6	62.1	91.0	19.3
≤45	433 (61.3)	64.3	57.9	90.8	20.1
≤50	445 (63.0)	65.8	54.7	90.3	19.9
≤55	459 (65.0)	68.1	54.7	90.6	21.1
≤60	465 (65.9)	68.9	53.7	90.5	21.2

^a^ False‐positive rate can be by calculated using the formula FPR = 1 − specificity.

**TABLE 2:** Distribution of NCS severity grading across the different timed Phalen’s test cutoff values in 706 hands diagnosed with CTS

Phalen time (s)	NCS severity					
	0‐normal (*N* = 95)	1‐minimal (*N* = 129)	2‐mild (*N* = 148)	3‐moderate (*N* = 249)	4‐severe (*N* = 73)	5‐extreme (*N* = 12)
≤10	3 (3.2)	18 (14.0)	11 (7.4)	39 (15.7)	12 (16.4)	5 (41.7)
≤15	10 (10.5)	29 (22.5)	30 (20.3)	65 (26.1)	19 (26.0)	5 (41.7)
≤20	14 (14.7)	36 (27.9)	43 (29.1)	94 (37.8)	32 (43.8)	5 (41.7)
≤25	17 (17.9)	43 (33.3)	58 (39.2)	118 (47.4)	42 (57.5)	5 (41.7)
≤30	28 (29.5)	50 (38.8)	71 (48.0)	140 (56.2)	48 (65.8)	5 (41.7)
≤35	32 (33.7)	57 (44.2)	77 (52.0)	145 (58.2)	49 (67.1)	5 (41.7)
≤40	36 (37.9)	61 (47.3)	85 (57.4)	159 (63.9)	54 (74.0)	5 (41.7)
≤45	40 (42.1)	69 (53.5)	88 (59.5)	173 (69.5)	58 (79.5)	5 (41.7)
≤50	43 (45.3)	70 (54.3)	91 (61.5)	177 (71.1)	59 (80.8)	5 (41.7)
≤55	43 (45.3)	74 (57.4)	94 (63.5)	183 (73.5)	59 (80.8)	6 (50.0)
≤60	44 (46.3)	75 (58.1)	94 (63.5)	186 (74.7)	59 (80.8)	7 (58.3)
Negative	51 (53.7)	54 (41.9)	54 (36.5)	63 (25.3)	14 (19.2)	5 (41.7)

## 4 DISCUSSION

This study found that shorter Phalen times correlated with higher specificity and PPV in predicting the presence of abnormal NCS results, but not NCS severity. However, the sensitivity of the low TPT cutoff values was poor, and even at 60 s, it was within the range of the previously reported sensitivities (Brüske et al., 2002). Nevertheless, a positive TPT at ≤10 s had a high specificity and low false‐positive rate (FPR = 1 − specificity, 3.2%), which indicate that it has value as a rule‐in test in clinical diagnoses. Higher cutoff values can be chosen based on false‐positive rates that would be tolerable for individual cases. The PPV remained above 90% for all cutoff values. PPV and NPV are influenced by disease prevalence. The prevalence of abnormal NCS in our study is consistent with that reported in the literature (Werner & Andary, 2011). Furthermore, the electrodiagnostic techniques and reference values used here are consistent with the recommendations by the American Academy of Neuromuscular and Electrodiagnostic Medicine (Werner & Andary, 2011).

Consistent with previous studies, (Nora et al., 2004) the TPT at all cutoff values was positive in a higher proportion of patients with moderate and severe NCS than in those with minimal and mild NCS; however, the correlation coefficient was negligible. Ansari et al. (2009) reported associations between the electrophysiological abnormalities and a positive Phalen test at 30 s, but not at 45 or 60 s. Others have suggested that the onset of symptoms within 30 s indicates severe involvement of the median nerve (Schroeder & Botte, 1996). Although it was not planned a priori, after dichotomizing the TPT into “≤30 s” and “>30 s,” NCS severity was statistically worse in the former (median score 3 and 2, respectively; *p* = .03). However, the clinical significance of this observation is unclear. Interestingly, patients with extreme NCS were nearly dichotomized into a group with TPT ≤ 10 s and a group with negative TPT, suggesting that some pain/sensory fibers below the threshold of detection by NCS may have been preserved in the former group.

This study did not examine correlations between the clinical severity of CTS and NCS severity, nor did it explore whether individual symptoms or signs (other than Phalen sign) predict abnormal NCS. Several studies have reported associations between clinical findings and abnormal NCS parameters; these findings were pain and diurnal paresthesia, (Ansari et al., 2009) hypalgesia in the median nerve distribution, weak thumb abduction, classic or probable hand symptom diagram, (D’Arcy & McGee, 2000) and severity of CTS sensorimotor symptoms and signs (Zeidman & Pandey, 2018).

The methodologies used in this study had several limitations. The lack of a control group may have increased the risk of overestimating the results. The Phalen test requires elbow flexion, which may also have confounded the TPT with symptoms emanating from ulnar neuropathy. To minimize this possibility, we preemptively excluded patients with clinical features suggestive of a coexisting ulnar neuropathy and used a stringent definition for a positive TPT, requiring that the triggered symptoms are limited to the median nerve distribution. We did not exclude patients with diabetes, hypothyroidism, and mild polyneuropathy restricted to lower limbs. These conditions commonly coexist with CTS, and excluding them limits the utility of the TPT.

In conclusion, a positive TPT at ≤10 s can be useful in predicting NCS abnormalities in patients with clinical CTS.

## CONFLICTS OF INTEREST

None of the authors have any conflict of interest to disclose.

## AUTHOR CONTRIBUTION

Mohammed H. Alanazy was involved in study design, data acquisition and interpretation, statistical analysis, and drafting the manuscript. Hana Albulaihe, Ziad Alhumayyd, Anas M. Albarrak, and Ahmad R. Abuzinadah were involved in data acquisition and interpretation, and reviewing the manuscript.

## ETHICAL APPROVAL

We confirm that we have read the journal's position on issues involved in ethical publication and affirm that this report is consistent with those guidelines.

### PEER REVIEW

The peer review history for this article is available at https://publons.com/publon/10.1002/brb3.2056.

## Supporting information

Supplementary Material

## Data Availability

The data that support the findings of this study are available from the corresponding author upon reasonable request.
