# Supplementary material for: A timed Phalen’s test predicts abnormal electrophysiology in carpal tunnel syndrome
Source: Brain Behav. 2021 Jan 29;11(4):e02056. doi: 10.1002/brb3.2056 (PMC8035481; doi:10.1002/brb3.2056)

Supplementary materials

Table S1. Diagnostic accuracy of right hand timed Phalen test in predicting abnormal NCS results in patients with CTS (N = 367 hands).

| Phalen time (seconds) | Hands, N(%) | Sensitivity (%) | Specificity (%) | Positive predictive value (%) | Negative predictive value (%) |
| --- | --- | --- | --- | --- | --- |
| ≤10 | 46 (12.5) | 14.1 | 97.9 | 97.8 | 14.3 |
| ≤15 | 80 (21.8) | 24.1 | 93.6 | 96.3 | 15.3 |
| ≤20 | 114 (31.1) | 33.8 | 87.2 | 94.7 | 16.2 |
| ≤25 | 142 (38.7) | 42.2 | 85.1 | 95.1 | 17.8 |
| ≤30 | 172 (46.9) | 50.3 | 76.6 | 93.6 | 18.5 |
| ≤35 | 184 (50.1) | 52.8 | 68.1 | 91.8 | 17.5 |
| ≤40 | 201 (54.8) | 57.5 | 63.8 | 91.5 | 18.1 |
| ≤45 | 220 (59.9) | 62.5 | 57.4 | 90.9 | 18.4 |
| ≤50 | 229 (62.4) | 64.7 | 53.2 | 90.4 | 18.1 |
| ≤55 | 236 (64.3) | 66.9 | 53.2 | 90.7 | 19.1 |
| ≤60 | 240 (65.4) | 67.8 | 51.1 | 90.4 | 18.9 |

Table S2. Diagnostic accuracy of left hand timed Phalen test in predicting abnormal NCS results in patients with CTS (N = 339 hands).

| Phalen time (seconds) | Hands, N(%) | Sensitivity (%) | Specificity (%) | Positive predictive value (%) | Negative predictive value (%) |
| --- | --- | --- | --- | --- | --- |
| ≤10 | 42 (12.4) | 13.7 | 95.8 | 95.2 | 15.5 |
| ≤15 | 78 (23.0) | 24.4 | 85.4 | 91.0 | 15.7 |
| ≤20 | 110 (32.4) | 35.1 | 83.3 | 92.7 | 17.5 |
| ≤25 | 141 (41.6) | 45.0 | 79.2 | 92.9 | 19.2 |
| ≤30 | 170 (50.1) | 52.6 | 64.6 | 90.0 | 18.3 |
| ≤35 | 181 (53.4) | 56.4 | 64.6 | 90.6 | 19.6 |
| ≤40 | 199 (58.7) | 61.9 | 60.4 | 90.5 | 20.7 |
| ≤45 | 213 (62.8) | 66.3 | 58.3 | 90.6 | 22.2 |
| ≤50 | 216 (63.7) | 67.0 | 56.3 | 90.3 | 22.0 |
| ≤55 | 223 (65.9) | 69.4 | 56.3 | 90.6 | 23.3 |
| ≤60 | 225 (66.4) | 70.1 | 56.3 | 90.7 | 23.7 |

Table S3. Right and left hand Phalen time statistics.

| Phalen Time (seconds) | Left hand (N=225) | Right hand (N=240) |
| --- | --- | --- |
| Mean (SD) | 23.4 (13.1) | 24.8 (14.1) |
| Range | 1 – 58 | 1 – 60 |
| 25^th^ percentile | 13 | 14 |
| 50^th^ percentile (Median) | 21 | 22 |
| 75^th^ percentile | 30 | 35 |

Table S4. Comparison between Phalen time in diabetics and non-diabetics per hand.

|  | Diabetes | N | Mean (SD) | P value |
| --- | --- | --- | --- | --- |
| Left hand Phalen time | No | 148 | 23.0 (12.5) | 0.51 |
|  | Yes | 77 | 24.2 (14.2) |  |
| Right hand Phalen time | No | 163 | 25.1 (14.0) | 0.57 |
|  | Yes | 77 | 24.0 (14.5) |  |

**Spearman correlations**

- Phalen time and NCS severity: Rho = – 0.13 (P=0.006)
- Left hand Phalen time and diabetes: Rho = –0.02 (P=0.82)
- Right hand Phalen time and diabetes: Rho = –0.05 (P=0.42)
- Age and Left hand Phalen time: Rho = –0.07 (P=0.36)
- Age and Right hand Phalen time: Rho = –0.12 (P=0.07)


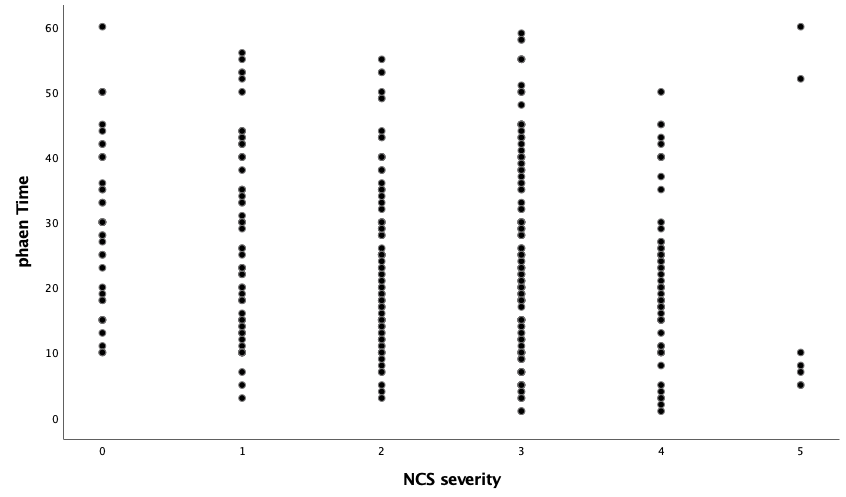


NCS severity scores in TPT ≤30 seconds vs >30 seconds


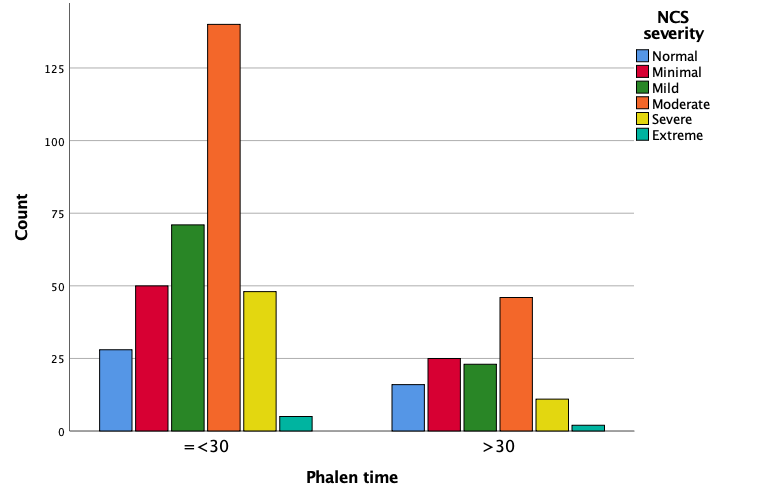

Supplement: Supplementary file 1 — Supplementary Material [file BRB3-11-e02056-s001.docx]
